# Supplementary material for: What Are The Core Symptoms of Antenatal Depression? A Study Using Patient Health Questionnaire-9 among Japanese Pregnant Women in the First Trimester
Source: Healthcare (Basel). 2023 May 20;11(10):1494. doi: 10.3390/healthcare11101494 (PMC10218321; doi:10.3390/healthcare11101494)
Supplement: Supplementary file 1 [file healthcare-11-01494-s001.zip › healthcare-2363949-supplementary.pdf]

Supplementary Table S1. Model fit indices as 1-factor model for Non-somatic domain of PHQ-9.

**PHQ-9**	χ^2	*df*	χ^2/df	**CFI**	**RMSEA**	**AIC**	**TLI**	**SRMR**
Non-somatic (excluding item 9 [suicidality])	50.483	5	10.16	0.929	0.155	3950.293	0.857	**0.048**

Note. PHQ-9, Patient health questionnaire 9; CFI, Comparative fit index; RMSEA, Root mean squared error of approximation; AIC, Akaike information Criteria; TLI, Tucker-Lewis index; SRMR, standardized Root Mean Square Residual.

Criteria for fit indices: CFI > 0.95 for excellent fit; RMSEA < 0.06 for good fit; TLI > 0.95 for excellent; fit; SRMR < 0.08 for good fit

Supplementary Table S2. Residual correlations and monotonicity.

PHQ-9		Residual correlations					scalability
Non somatic subscale		1:	2:	6:	7:	8:	
1:	Loss of interest	—					0.584
2:	Depressed mood	.043	—				0.563
6:	Self-esteem	−.065	−.034	—			0.561
7:	Poor concentration	.031	−.083	−.028	—		0.541
8:	Psychomotor	−.052	−.056	−.069	.088	—	0.399

Note. PHQ-9, Patient health questionnaire 9; Scalabilities, Mokken scalability coefficients Hi
